# Glucose tolerance phenotypes and risks of incident first-ever stroke and all-cause mortality in low-income rural Chinese adults: a population-based prospective cohort study

**DOI:** 10.3389/fendo.2026.1924067

**Published:** 2026-09-17

**Authors:** Lei Zhang, Xiao Li, Juan Hao, Chenxi Fan, Jun Tu, Jinghua Wang, Xianjia Ning, Yaogang Wang, Yan Li

**Affiliations:** 1 School of Public Health, Tianjin Medical University, Tianjin, China; 2 Department of Neurology, Tianjin Medical University General Hospital, Tianjin, China; 3 Institute of Clinical Epidemiology & Evidence-Based Medicine, Tianjin Jizhou People's Hospital, The Fifth Affiliated Hospital of Tianjin University of Traditional Chinese Medicine, Tianjin, China; 4 Laboratory of Epidemiology, Tianjin Neurological Institute, Tianjin, China; 5 Tianjin Neurological Institute, Key Laboratory of Post-Neuroinjury Neuro-repair and Regeneration in Central Nervous System, Ministry of Education and Tianjin City, Tianjin, China; 6 School of Integrative Medicine, Public Health Science and Engineering College, Tianjin University of Traditional Chinese Medicine, Tianjin, China

**Keywords:** all-cause mortality, glucose tolerance status, incident stroke, prospective cohort study, rural population

## Abstract

**Background and objective:**

Impaired fasting glucose (IFG) and impaired glucose tolerance (IGT) are common intermediate hyperglycaemic states, yet most cohorts have compared diabetes with non-diabetes, or have relied on fasting glucose alone, so the stroke risk gradient across the full glucose tolerance spectrum and the relative prognostic value of fasting versus postload glycaemia remain unresolved. Evidence is particularly scarce for low-income rural Chinese populations, in whom vascular risk factors cluster while chronic-disease management and screening resources are limited. This study aimed to investigate the associations of glucose tolerance status with incident first-ever post-baseline stroke and all-cause mortality among low-income rural adults in China.

**Methods:**

This population-based prospective cohort study included 2,087 adults from a low-income rural community in northern China who underwent a baseline examination including a 75-g oral glucose tolerance test (OGTT) in 2019. Among them, 2,057 participants with valid baseline examination dates were included in the time-to-event analyses, contributing 13,074.9 person-years for stroke and 13,477.4 person-years for mortality over a mean follow-up of 6.4 years. Baseline glucose tolerance status was classified as normal glucose tolerance (NGT), IFG, IGT, or diabetes according to the WHO/IDF criteria. Incident first-ever post-baseline stroke was defined as the first stroke event occurring after the 2019 baseline examination; participants with a stroke before baseline were excluded from this analysis, and all-cause mortality was recorded during follow-up. Incidence rates were calculated per 1,000 person-years and were additionally standardised for age and sex by the direct method. Cox proportional hazards regression models were used to estimate HRs and 95% CIs. The primary multivariable model (Model 2) was adjusted for sex, age group, smoking, drinking and eGFR; metabolic syndrome was added only in Model 3 and examined separately, because it shares glycaemic components with the exposure. In addition, fasting glucose and 2-h postload glucose were analysed as continuous variables in separate models, so that fasting and postload glycaemia could be compared without inducing collinearity. Proportional hazards were assessed using scaled Schoenfeld residuals, and multiple-imputation, Fine–Gray competing-risk and prespecified subgroup analyses were performed as sensitivity analyses.

**Results:**

During follow-up, 121 participants developed incident first-ever post-baseline stroke and 68 died from any cause, corresponding to overall incidence rates of 9.25 and 5.05 per 1,000 person-years, respectively. For incident stroke, the incidence rates were 7.55, 5.40, 12.74, and 14.06 per 1,000 person-years in the NGT (n = 1,324; 63.4%), IFG (n = 86; 4.1%), IGT (n = 449; 21.5%), and diabetes (n = 228; 10.9%) groups, respectively; for all-cause mortality the corresponding rates were 4.68, 1.77, 6.27 and 6.05 per 1,000 person-years. In crude analyses, both IGT (HR 1.69, 95% CI 1.11–2.55, *P* = 0.013) and diabetes (HR 1.85, 95% CI 1.12–3.06, *P* = 0.016) were associated with a higher risk of incident first-ever stroke compared with NGT. In the primary multivariable model (Model 2), both IGT (HR 1.58, 95% CI 1.03–2.41, *P* = 0.036) and diabetes (HR 1.69, 95% CI 1.02–2.81, *P* = 0.043) remained associated with incident stroke, whereas IFG did not (HR 0.78, 95% CI 0.25–2.51, *P* = 0.682). Adding metabolic syndrome (Model 3) attenuated both estimates and rendered diabetes non-significant (IGT: HR 1.55, 95% CI 1.01–2.39, *P* = 0.046; diabetes: HR 1.63, 95% CI 0.96–2.79, *P* = 0.071). No glucose tolerance subtype was significantly associated with all-cause mortality in any model. In continuous analyses modelled separately, both fasting glucose (HR 1.18 per 1 mmol/L, 95% CI 1.01–1.38, *P* = 0.035) and 2-h postload glucose (HR 1.07 per 1 mmol/L, 95% CI 1.02–1.13, *P* = 0.008) were associated with incident stroke; per standard deviation the postload association was the stronger of the two (HR 1.23, 95% CI 1.05–1.42 versus HR 1.17, 95% CI 1.01–1.35). Neither measure was associated with mortality.

**Conclusion:**

Three conclusions follow. First, in this specific setting — a low-income rural Chinese community with clustered vascular risk factors and limited screening capacity — both IGT and diabetes were associated with an increased risk of incident first-ever stroke, whereas isolated IFG was not. Second, postload glycaemia carried more prognostic information for stroke than fasting glycaemia: the postload association was the stronger of the two per standard deviation, and only the postload phenotype (IGT) showed an excess risk in the categorical analysis, while the continuous fasting association appeared to be driven largely by participants in the diabetes range rather than by isolated fasting hyperglycaemia. Third, for grassroots public-health practice these findings identify IGT as a candidate phenotype for earlier vascular risk assessment; whether adding postload measurement to existing screening improves outcomes remains to be tested directly.

## 1 Introduction

Stroke remains one of the leading causes of death and long-term disability worldwide and continues to impose a substantial public health burden, particularly in low- and middle-income countries (1, 2). In China, the burden of stroke is especially heavy. National estimates indicate that among adults aged 40 years and older, stroke accounted for approximately 3.4 million incident cases, 17.8 million prevalent cases, and 2.3 million deaths in 2020 (3). This national burden, however, is borne disproportionately by rural and socioeconomically disadvantaged communities. In the same low-income rural region of northern China from which the present cohort is drawn, the age-standardised incidence of first-ever stroke reached 10.1 per 1,000 person-years over 27 years of surveillance, roughly threefold the contemporaneous rates reported from Japanese and Western community cohorts, and the annual increase in first-ever stroke incidence between 1992 and 2012 was 6.5% (4). Low income and low educational attainment act independently and jointly on stroke incidence in rural Chinese communities (5). Three features of these communities compound the risk: vascular risk factors cluster and are poorly controlled, long-term chronic-disease management is weak, and screening resources are scarce, so that metabolic abnormalities are frequently detected only after a vascular event has occurred (4, 5).

At the same time, disorders of glucose metabolism have become increasingly prevalent and a major threat to population health. In addition to overt diabetes, intermediate states of dysglycaemia — IFG and IGT — are highly prevalent and increasingly recognized as clinically important conditions rather than merely transitional metabolic abnormalities (6). The vascular hazard attached to these three states, however, is not equivalent, and the literature separates fairly cleanly into three strands. (i) Overt diabetes is an established cause of excess stroke and premature death, and recent national Chinese data confirm that diabetes shortens life expectancy (7). (ii) Prediabetes as an undifferentiated category carries a modest excess risk of all-cause mortality and cardiovascular disease (8), and meta-analytic evidence indicates a higher future risk of stroke (9). (iii) Critically, when prediabetes is disaggregated, the excess risk does not distribute evenly: the DECODE Study and the related Finnish analyses showed that 2-h postload glucose predicts cardiovascular mortality better than fasting glucose (10, 11), and the Da Qing studies showed that IGT is not only a precursor of diabetes but is itself associated with subsequent cardiovascular events and mortality across decades of follow-up, and that lifestyle intervention in individuals with IGT reduces those long-term risks (12–14). Chinese community cohorts have reported associations of fasting glucose with incident stroke (15) and all-cause mortality (16), but have rarely been able to place fasting and postload measures side by side in the same population.

Against this background, three specific gaps motivated the present study. First, most previous studies have used a dichotomous exposure (diabetes versus non-diabetes) or have treated fasting glucose as a single continuous measure, without separating IFG, IGT and diabetes as distinct metabolic phenotypes (8, 9, 15, 16); as a result, the stroke risk gradient across the full spectrum of glucose tolerance remains insufficiently characterised. Second, although the DECODE Study and subsequent analyses suggest that 2-h postload glucose carries greater prognostic value than fasting glucose (10, 11), this remains contested (15, 16), and head-to-head comparative evidence from community-based Chinese cohorts that have actually performed an OGTT is scarce. Third, socioeconomic context plausibly modifies the health consequences of dysglycaemia: low-income rural populations in China have lower educational attainment, a higher prevalence of uncontrolled vascular risk factors and less access to prevention and long-term care (4, 5), yet no prospective cohort has specifically examined glucose tolerance phenotypes in relation to both incident stroke and all-cause mortality in such a population.

Accordingly, the primary objective of this study was to compare the risk of incident first-ever post-baseline stroke across the four glucose tolerance phenotypes (NGT, IFG, IGT, diabetes) in a low-income rural Chinese cohort. The secondary objectives were to examine the same phenotypes in relation to all-cause mortality, and to compare fasting and 2-h postload glucose as continuous predictors of both outcomes. We prespecified three hypotheses: (i) IGT is associated with a greater increase in first-ever stroke risk than IFG in this population; (ii) 2-h postload glucose is a stronger predictor of stroke than fasting glucose; and (iii) glucose tolerance status is not associated with short- to mid-term all-cause mortality.

To test these hypotheses we used a population-based prospective cohort with a 2019 baseline examination that included a 75-g OGTT, approximately six years of subsequent follow-up for stroke and death, and four-category stratification of baseline glucose tolerance. The design, measurements and analytical strategy are described below.

## 2 Methods

### 2.1 Study design and study population

This population-based prospective cohort study was conducted in a low-income rural community in northern China. Baseline data were collected in 2019 through community-based health examinations and structured interviews. The study was reported in accordance with the STROBE statement for observational studies (17).

The source population comprised the permanent residents of the Tianjin Brain Study surveillance area, a group of administrative villages in Jizhou District, Tianjin, in which stroke events and deaths have been monitored continuously since 1985 (4). The community is agricultural: about 95% of adult residents are farmers whose principal income derives from grain production, and reported annual per capita income in this population was below 100 USD in 1991 and below 2,000 USD in 2015, placing it well below the contemporaneous national rural average (4, 5). Educational attainment is correspondingly low — 16.5% of the present cohort had no formal schooling and a further 40.7% had six years or less — and few residents were covered by national medical insurance before 2008 (4). Primary care is delivered through a three-tier village doctor–township health centre–district hospital system, and the district has no routine community programme for oral glucose tolerance testing; in practice, glucose abnormalities are usually detected only through fasting measurement at opportunistic contacts. These characteristics define the operational sense in which the cohort is described as ‘low-income rural’ throughout this paper, and they also indicate the setting to which the findings most directly extrapolate: rural northern Chinese communities with a comparable income level, educational profile and primary-care infrastructure.

Participants were eligible if they had attended the 2019 baseline examination with a valid 75-g OGTT and had follow-up information available. All 2,087 participants included in the baseline descriptive analyses had complete fasting and 2-h postload glucose measurements and could therefore be assigned a glucose tolerance category, and none had a stroke documented before the baseline examination; no participant was consequently excluded at either of these steps. A further 30 participants had no valid baseline examination date and could not contribute person-time, leaving 2,057 participants (NGT 1,305; IFG 85; IGT 441; diabetes 226) for the time-to-event analyses of both incident first-ever post-baseline stroke and all-cause mortality; because no participant had a pre-baseline stroke, the two outcomes share an identical analytic sample. Complete-case multivariable models additionally excluded participants with missing covariates; the numbers are given in the footnotes to the corresponding tables. The full participant flow is shown in **Supplementary Figure 1**, and baseline characteristics of included versus excluded participants are compared in **Supplementary Table 1**.

The study protocol was approved by the Ethics Committee of Tianjin Medical University General Hospital (Approval No. IRB2018-100-02), and all participants provided written informed consent at enrolment. Full ethical statements are provided in the Declarations.

### 2.2 Data collection

Baseline information was collected by trained investigators using standardized questionnaires, physical examinations, and laboratory testing. Demographic and lifestyle variables included sex, age, education, smoking, drinking, regular physical activity, and snoring. Anthropometric measurements included height and weight, and BMI was calculated as weight in kilograms divided by height in meters squared. Blood pressure was measured at baseline according to the field examination protocol.

All participants underwent a standard 75-g OGTT at the baseline examination. Participants were instructed to fast for at least 10 hours overnight (and for no more than 16 hours) and to avoid alcohol and vigorous exercise on the preceding day. After the fasting venous sample was drawn, 75 g of anhydrous glucose dissolved in 300 mL of water was ingested within 5 minutes, and a second venous sample was drawn exactly 120 minutes after the first sip. Participants remained seated at rest in the examination room, and did not eat, smoke or drink anything other than water, for the duration of the test. Plasma glucose was measured on the day of collection by the hexokinase method; the same analytical platform, reagent supplier and calibration protocol were used for the entire baseline survey. Internal quality-control specimens at two concentrations were run with every batch, and the laboratory participated in the national external quality assessment scheme of the National Centre for Clinical Laboratories. Fasting and 2-h postload glucose were available for every participant included in the analysis, with no missing values. TC, TG, HDL-C, LDL-C, serum creatinine and hs-CRP were measured on the same fasting sample using the same platform.

### 2.3 Definitions of study variables

#### 2.3.1 Glucose tolerance status

The main exposure was baseline glucose tolerance status. Participants were classified into four groups according to the WHO/IDF criteria for diabetes and intermediate hyperglycaemia (18), using the following cut-offs (venous plasma glucose): NGT, fasting glucose <6.1 mmol/L and 2-h postload glucose <7.8 mmol/L; IFG, fasting glucose 6.1–6.9 mmol/L and 2-h postload glucose <7.8 mmol/L; IGT, fasting glucose <7.0 mmol/L and 2-h postload glucose 7.8–11.0 mmol/L; and diabetes, fasting glucose ≥7.0 mmol/L or 2-h postload glucose ≥11.1 mmol/L or a documented previous diagnosis of diabetes. Participants meeting the diabetes criteria were assigned to the diabetes group irrespective of any concurrent IFG or IGT pattern; participants meeting both the IFG and the IGT thresholds were classified as IGT, since postload status was the phenotype of primary interest.

#### 2.3.2 Renal function

eGFR was calculated from serum creatinine using the CKD-EPI creatinine equation (19).

#### 2.3.3 Body mass index

BMI was categorized according to the Chinese adult criteria as normal/underweight (<24.0 kg/m²), overweight (24.0–27.9 kg/m²), and obesity (≥28.0 kg/m²) (20).

#### 2.3.4 Metabolic syndrome

Metabolic syndrome was defined according to the harmonized joint interim statement criteria. Participants were considered to have metabolic syndrome if at least three of the following components were present: elevated waist circumference using Chinese-specific thresholds, elevated TG, reduced HDL-C, elevated blood pressure or antihypertensive treatment, and elevated fasting glucose or previously diagnosed diabetes. Because the last of these components is defined by the same fasting glycaemic information that partly defines the exposure, metabolic syndrome was deliberately excluded from the primary multivariable model and entered only in a secondary model (see section 2.5).

#### 2.3.5 Lifestyle and socio-demographic covariates

Smoking status was classified as current (having smoked at least one cigarette per day for one year or longer and still smoking at the baseline interview), former (having met that criterion but having stopped for at least one year before baseline), or never. Drinking status was classified using the same temporal structure: current (consuming any alcoholic beverage at least once per week for one year or longer and still drinking at baseline), former (previously meeting that criterion but abstinent for at least one year), or never. Regular physical activity was defined as at least 30 minutes of moderate leisure-time or occupational physical activity on at least five days per week (4); because almost all participants performed agricultural manual labour, this variable captures deliberate additional activity rather than total energy expenditure. Education was recorded as completed years of formal schooling and grouped as 0 years, 1–6 years and >6 years. Snoring was recorded as habitual snoring on most nights, as reported by the participant or a co-resident.

### 2.4 Outcome ascertainment

The primary outcome was incident first-ever post-baseline stroke, and the secondary outcome was all-cause mortality. Stroke was defined, following WHO MONICA criteria (21), as rapidly developing clinical signs of focal or global disturbance of cerebral function lasting more than 24 hours or leading to death, with no apparent cause other than vascular origin. Ischaemic stroke (thrombotic infarction, cardioembolic stroke or lacunar infarction) and haemorrhagic stroke (intracerebral or subarachnoid haemorrhage) were both counted as events, and cases that could not be assigned to either category were recorded as undetermined; the numbers by subtype are given in section 3.3. Transient ischaemic attacks were excluded, as were silent infarcts detected on imaging in the absence of clinical symptoms; participants with a prior transient ischaemic attack who subsequently sustained a qualifying stroke were counted as incident cases.

Events were ascertained through the three-tier surveillance system that has operated in this community since 1985 (4): village doctors report suspected stroke events and all deaths to the township health centre, which forwards them to the district hospital and to the study team. Case ascertainment was supplemented by annual review of hospital admission and discharge records, the district death register and residents’ medical insurance claim records, and by scheduled follow-up contact with participants or their family members. Every reported event was then adjudicated by two study neurologists blinded to baseline glucose tolerance status, who reviewed the clinical record and, where available, the CT or MRI report; disagreements were resolved by a third senior neurologist. Imaging confirmation was available for the majority of the incident events. The date of stroke onset was taken as the date of symptom onset recorded in the clinical record; where that was unavailable, the date of first medical contact for the event was used. Deaths were identified from the district death register and confirmed with the family; the date of death was taken from the death certificate.

Stroke events occurring before baseline were excluded from the incident stroke analysis, and only the first post-baseline event was retained for time-to-event analyses. Recurrent events were therefore not analysed, and participants were excluded from the stroke analysis only on the basis of a stroke history documented at the baseline examination. All-cause mortality was defined as death from any cause during follow-up.

Person-time was calculated from the date of the 2019 baseline examination to the earliest of three censoring events. For the stroke analysis, follow-up ended at the date of incident first-ever stroke (event), the date of death without a preceding stroke (censored), or the common end-of-follow-up date date for participants who were alive and stroke-free, including those lost to follow-up, who were censored at the date of last known contact. For the mortality analysis, follow-up ended at the date of death (event) or at the same end-of-follow-up or last-contact date (censored); an intervening stroke did not censor follow-up for mortality. This is why person-time differs between the two outcome analyses (13,074.9 versus 13,477.4 person-years) despite an identical analytic sample.

### 2.5 Statistical analysis

Continuous variables are presented as mean ± standard deviation or median (interquartile range), as appropriate, and categorical variables as number (percentage). Baseline characteristics were summarized for the overall population and compared across glucose tolerance categories using one-way analysis of variance for normally distributed continuous variables, the Kruskal–Wallis test for skewed continuous variables, and the chi-square test for categorical variables. Where the overall test was significant, pairwise *post-hoc* comparisons were performed: the Student–Newman–Keuls procedure for normally distributed continuous variables, Dunn’s test with Bonferroni adjustment for skewed variables, and pairwise chi-square tests with Bonferroni adjustment for the six between-group contrasts of categorical variables. In the baseline comparison by glucose tolerance status, groups not sharing a superscript letter differ at the Bonferroni-adjusted two-sided α of 0.0083.

Incidence rates of incident first-ever stroke and all-cause mortality were calculated as the number of events divided by person-years of follow-up and expressed per 1,000 person-years. Because the four glucose tolerance groups differed substantially in age, incidence rates were additionally standardised for age and sex by the direct method using the WHO world standard population, with the age strata 45–59, 60–69 and ≥70 years. Kaplan–Meier curves were used to describe stroke-free survival and overall survival across glucose tolerance groups, and between-group differences were assessed using the log-rank test.

Cox proportional hazards regression models were used to estimate HRs and 95% CIs, with NGT as the reference category. Three nested models are reported. Model 1 is unadjusted. Model 2, the primary adjusted model, includes sex, age group, smoking status, drinking status and eGFR. Model 3 adds metabolic syndrome. Covariates for Model 2 were selected *a priori* on the basis of a causal diagram in which age, sex and smoking and drinking behaviour are common causes of both dysglycaemia and stroke, and in which renal function is a shared marker of microvascular injury; blood pressure, BMI and lipid fractions were deliberately not included, because in the assumed causal structure they lie on the pathway from insulin resistance to cerebrovascular events and adjusting for them would block part of the effect of interest. Metabolic syndrome was separated into Model 3 because its diagnostic definition incorporates elevated fasting glucose or diagnosed diabetes and therefore overlaps conceptually with the exposure; multicollinearity was assessed by variance inflation factors from the corresponding linear models and by comparison of the standard errors of the exposure coefficients across Models 2 and 3.

The proportional hazards assumption was examined for every covariate and for the exposure by testing the correlation between scaled Schoenfeld residuals and log-transformed survival time (global and variable-specific tests), by adding exposure-by-log(time) interaction terms to the models, and by visual inspection of the log(−log(survival)) plots. Results are reported in section 3.7.

To further assess the prognostic value of continuous glycaemic indicators, fasting glucose and 2-h postload glucose were each entered into a separate adjusted Cox model — never simultaneously — because the two measures are strongly correlated and joint entry would induce collinearity and render the individual coefficients uninterpretable. For the same reason, metabolic syndrome was omitted from these models, since its fasting-glucose component is collinear with the fasting-glucose exposure. HRs are reported per 1 mmol/L increment, and additionally per 1 standard deviation of the cohort distribution, so that the two measures can be compared on a common scale.

Complete-case analysis was used for the primary models. Three sets of sensitivity analyses were prespecified. First, missing covariate and exposure data were addressed by multiple imputation by chained equations, with imputed datasets generated under a missing-at-random assumption using predictive mean matching for continuous variables and logistic or multinomial models for categorical variables, including all model covariates, the Nelson–Aalen cumulative hazard estimate and the event indicator in the imputation model; estimates were pooled by Rubin’s rules and compared with the complete-case estimates. Second, because death precludes subsequent observation of stroke, the cause-specific Cox model for incident stroke was supplemented by a Fine–Gray subdistribution hazard model treating death without stroke as a competing event. Third, prespecified subgroup analyses were conducted by age (<60 versus ≥60 years), sex, and presence of metabolic syndrome, with effect modification assessed by likelihood ratio tests of multiplicative interaction terms between glucose tolerance status and each stratifying variable.

No formal sample size calculation was performed before this analysis, since the cohort size was fixed by the underlying surveillance population. We therefore quantified the effect sizes the study was able to detect. Using Schoenfeld’s formula for the log-rank test at a two-sided α of 0.05 and 80% power, the 95 stroke events accrued in the NGT and IGT groups of the complete-case sample were sufficient to detect an HR of 1.94 or greater for IGT versus NGT; the corresponding minimum detectable HRs were 2.40 for diabetes (81 events) and 4.34 for IFG (64 events). For all-cause mortality, the minimum detectable HRs were 2.36, 3.12 and 6.40 for IGT, diabetes and IFG respectively. The observed IGT–stroke HR of 1.58 and diabetes–stroke HR of 1.69 both lie below these thresholds, corresponding to approximately 49% and 39% power.

All statistical analyses were performed using SPSS version 25.0 (IBM Corp., Armonk, NY, USA), except the Fine–Gray competing-risk models, which were fitted in R using the cmprsk package. All tests were two-sided, and a *P* value <0.05 was considered statistically significant.

## 3 Results

### 3.1 Baseline characteristics of the study population

A total of 2,087 participants were included in the baseline analysis, including 926 men and 1,161 women. The baseline characteristics of the study population are presented in **Table 1**, with sex-stratified distributions shown for descriptive purposes. Overall, this cohort consisted mainly of middle-aged and older adults from a low-income rural community, with a substantial burden of overweight or obesity, hypertension, dyslipidaemia, metabolic syndrome, and diagnosed diabetes.

**Table 1:** Baseline characteristics by sex.

Variable	Total (n = 2,087)	Male (n = 926)	Female (n = 1,161)	P value
Age, years	63.43 ± 7.88	64.53 ± 7.77	62.54 ± 7.86	<0.001
Age group, n (%)				<0.001
45–59	643 (30.8)	218 (23.5)	425 (36.6)	
60–69	1,055 (50.6)	506 (54.6)	549 (47.3)	
≥70	389 (18.6)	202 (21.8)	187 (16.1)	
Education, n (%)				<0.001
0 years	345 (16.5)	49 (5.3)	296 (25.5)	
1–6 years	849 (40.7)	394 (42.5)	455 (39.2)	
>6 years	893 (42.8)	483 (52.2)	410 (35.3)	
BMI, kg/m²	25.61 ± 3.68	25.03 ± 3.39	26.07 ± 3.83	<0.001
BMI category, n (%)				<0.001
Normal/underweight (<24.0)	704 (33.8)	368 (39.8)	336 (29.0)	
Overweight (24.0–27.9)	889 (42.7)	384 (41.5)	505 (43.6)	
Obesity (≥28.0)	489 (23.5)	173 (18.7)	316 (27.3)	
Hypertension, n (%)	1546 (74.3)	706 (76.5)	840 (72.5)	0.046
Diagnosed diabetes, n (%)	228 (10.9)	103 (11.1)	125 (10.8)	0.850
Dyslipidemia, n (%)	787 (37.7)	308 (33.3)	479 (41.3)	<0.001
Metabolic syndrome, n (%)	948 (45.4)	305 (32.9)	643 (55.4)	<0.001
Smoking status, n (%)				<0.001
Current	526 (25.4)	485 (53.1)	41 (3.6)	
Former	203 (9.8)	196 (21.4)	7 (0.6)	
Never	1,338 (64.7)	233 (25.5)	1,105 (95.8)	
Drinking status, n (%)				<0.001
Current	425 (20.5)	408 (44.6)	17 (1.5)	
Former	96 (4.6)	91 (9.9)	5 (0.4)	
Never	1,551 (74.9)	416 (45.5)	1,135 (98.1)	
Regular physical activity, n (%)	1,282 (61.4)	552 (59.6)	730 (62.9)	0.140
Snoring, n (%)	1,060 (50.8)	484 (52.3)	576 (49.6)	0.235
SBP, mmHg	149.08 ± 19.71	149.91 ± 19.74	148.42 ± 19.66	0.088
DBP, mmHg	85.18 ± 10.75	87.50 ± 10.74	83.34 ± 10.41	<0.001
Fasting glucose, mmol/L	5.54 ± 0.92	5.56 ± 0.97	5.53 ± 0.88	0.430
2-h postload glucose, mmol/L	7.28 ± 2.87	6.97 ± 2.91	7.52 ± 2.81	<0.001
TC, mmol/L	5.14 ± 0.87	4.94 ± 0.80	5.31 ± 0.90	<0.001
TG, mmol/L	1.26 (0.90–1.83)	1.08 (0.79–1.59)	1.41 (1.00–1.97)	<0.001
HDL-C, mmol/L	1.43 ± 0.45	1.44 ± 0.50	1.42 ± 0.41	0.306
LDL-C, mmol/L	3.12 ± 0.87	2.96 ± 0.81	3.26 ± 0.89	<0.001
eGFR, mL/min/1.73 m²	87.90 ± 11.88	87.91 ± 11.57	87.89 ± 12.12	0.972
hs-CRP, mg/L	1.07 (0.62–2.16)	1.01 (0.57–2.06)	1.12 (0.65–2.26)	0.027

Continuous variables are presented as mean ± SD or median (IQR); categorical variables as n (%) with non-missing denominators. Continuous variables were compared between sexes using Student’s *t*-test (normally distributed) or the Mann–Whitney *U* test (skewed); categorical variables using the chi-square test. Missing data: hypertension 6, BMI 5, smoking 20, drinking 15, snoring 1, dyslipidemia 1, blood pressure 11, hs-CRP 250. Abbreviations: BMI, body mass index; SBP/DBP, systolic/diastolic blood pressure; TC, total cholesterol; TG, triglycerides; HDL-C/LDL-C, high-/low-density lipoprotein cholesterol; eGFR, estimated glomerular filtration rate; hs-CRP, high-sensitivity C-reactive protein.

### 3.2 Baseline characteristics according to glucose tolerance status

The baseline characteristics of the study population according to glucose tolerance status are shown in **Table 2**. Of the 2,087 participants, 1,324 (63.4%) were classified as having NGT, 86 (4.1%) as IFG, 449 (21.5%) as IGT, and 228 (10.9%) as diabetes. The IFG group is small in absolute terms, and this constrains the precision of every IFG estimate reported below; with 66 stroke events in the NGT and IFG groups combined, the study had 80% power only for an HR of 4.34 or greater, so the absence of a significant IFG–stroke association should be read as an uninformative result rather than as evidence of no association.

**Table 2:** Baseline characteristics according to glucose tolerance status.

Variable	Total (n = 2,087)	NGT (n = 1,324; 63.4%)	IFG (n = 86; 4.1%)	IGT (n = 449; 21.5%)	Diabetes (n = 228; 10.9%)	P value
Sex, n (%)						0.109
Male	926 (44.4)	604 (45.6)	42 (48.8)	177 (39.4)	103 (45.2)	
Female	1161 (55.6)	720 (54.4)	44 (51.2)	272 (60.6)	125 (54.8)	
Age, years	63.43 ± 7.88	62.65 ± 7.82^b^	63.49 ± 8.51^ab^	64.88 ± 7.64^a^	65.05 ± 7.87^a^	<0.001
Age group, n (%)						<0.001
45–59	643 (30.8)	456 (34.4)^b^	25 (29.1)^ab^	109 (24.3)^a^	53 (23.2)^a^	
60–69	1055 (50.6)	663 (50.1)	43 (50.0)	231 (51.4)	118 (51.8)	
≥70	389 (18.6)	205 (15.5)	18 (20.9)	109 (24.3)	57 (25.0)	
Education level, n (%)						0.007
0 years	345 (16.5)	200 (15.1)^a^	18 (20.9)^ab^	88 (19.6)^b^	39 (17.1)^ab^	
1–6 years	849 (40.7)	516 (39.0)	32 (37.2)	199 (44.3)	102 (44.7)	
>6 years	893 (42.8)	608 (45.9)	36 (41.9)	162 (36.1)	87 (38.2)	
BMI, kg/m²	25.61 ± 3.68	25.23 ± 3.63^a^	26.07 ± 3.59^ab^	26.03 ± 3.69^a^	26.76 ± 3.62^b^	<0.001
BMI category, n (%)						<0.001
Normal/underweight	704 (33.8)	500 (37.9)^b^	26 (30.2)^ab^	127 (28.4)^a^	51 (22.4)^a^	
Overweight	889 (42.7)	550 (41.6)	37 (43.0)	203 (45.4)	99 (43.4)	
Obesity	489 (23.5)	271 (20.5)	23 (26.7)	117 (26.2)	78 (34.2)	
Hypertension, n (%)						<0.001
Yes	1546 (74.3)	901 (68.3)^b^	76 (89.4)^a^	370 (82.6)^a^	199 (87.3)^a^	
No	535 (25.7)	419 (31.7)	9 (10.6)	78 (17.4)	29 (12.7)	
Diagnosed diabetes, n (%)						<0.001
Yes	228 (10.9)	0 (0.0)^a^	0 (0.0)^a^	0 (0.0)^a^	228 (100.0)^b^	
No	1859 (89.1)	1324 (100.0)	86 (100.0)	449 (100.0)	0 (0.0)	
Dyslipidemia, n (%)						<0.001
Yes	787 (37.7)	447 (33.8)^b^	34 (39.5)^ab^	192 (42.9)^a^	114 (50.0)^a^	
No	1299 (62.3)	877 (66.2)	52 (60.5)	256 (57.1)	114 (50.0)	
Metabolic syndrome, n (%)						<0.001
Yes	948 (45.4)	461 (34.8)^b^	62 (72.1)^a^	249 (55.5)^c^	176 (77.2)^a^	
No	1139 (54.6)	863 (65.2)	24 (27.9)	200 (44.5)	52 (22.8)	
Smoking status, n (%)						0.106
Current	526 (25.4)	352 (26.9)	25 (29.1)	88 (19.8)	61 (27.0)	
Former	203 (9.8)	128 (9.8)	6 (7.0)	48 (10.8)	21 (9.3)	
Never	1338 (64.7)	830 (63.4)	55 (64.0)	309 (69.4)	144 (63.7)	
Drinking status, n (%)						0.463
Current	425 (20.5)	278 (21.2)	15 (17.6)	78 (17.4)	54 (23.7)	
Former	96 (4.6)	64 (4.9)	4 (4.7)	18 (4.0)	10 (4.4)	
Never	1551 (74.9)	970 (73.9)	66 (77.6)	351 (78.5)	164 (71.9)	
Regular physical activity, n (%)						0.368
Yes	1282 (61.4)	797 (60.2)	51 (59.3)	286 (63.7)	148 (64.9)	
No	805 (38.6)	527 (39.8)	35 (40.7)	163 (36.3)	80 (35.1)	
Snoring, n (%)						0.636
Yes	1060 (50.8)	661 (50.0)	43 (50.0)	232 (51.7)	124 (54.4)	
No	1026 (49.2)	662 (50.0)	43 (50.0)	217 (48.3)	104 (45.6)	
SBP, mmHg	149.08 ± 19.71	146.05 ± 19.26^c^	156.02 ± 19.22^ab^	152.19 ± 19.15^a^	157.87 ± 19.40^b^	<0.001
DBP, mmHg	85.18 ± 10.75	84.37 ± 10.42^a^	89.09 ± 10.84^b^	85.46 ± 11.32^a^	87.89 ± 10.73^b^	<0.001
Fasting glucose, mmol/L	5.54 ± 0.92	5.20 ± 0.43^a^	6.35 ± 0.22^b^	5.62 ± 0.54^c^	7.10 ± 1.63^d^	<0.001
2-h postload glucose, mmol/L	7.28 ± 2.87	5.78 ± 1.18^a^	6.02 ± 1.30^a^	8.99 ± 0.89^b^	13.04 ± 3.55^c^	<0.001
TC, mmol/L	5.14 ± 0.87	5.08 ± 0.84^b^	5.14 ± 0.90^ab^	5.26 ± 0.90^a^	5.27 ± 0.99^a^	<0.001
TG, mmol/L	1.26 (0.90–1.83)	1.17 (0.86–1.67)^a^	1.26 (0.90–1.87)^ab^	1.40 (0.99–1.98)^b^	1.58 (1.06–2.30)^c^	<0.001
HDL-C, mmol/L	1.43 ± 0.45	1.46 ± 0.46^b^	1.42 ± 0.51^ab^	1.39 ± 0.42^a^	1.32 ± 0.43^a^	<0.001
LDL-C, mmol/L	3.12 ± 0.87	3.08 ± 0.83^a^	3.13 ± 0.83^ab^	3.22 ± 0.93^b^	3.20 ± 0.98^ab^	0.010
eGFR, mL/min/1.73 m²	87.90 ± 11.88	88.12 ± 11.65	89.81 ± 11.14	86.87 ± 12.22	87.94 ± 12.64	0.110
hs-CRP, mg/L	1.07 (0.62–2.16)	0.96 (0.57–1.87)^b^	1.01 (0.64–2.23)^ab^	1.24 (0.71–2.53)^a^	1.61 (0.79–3.21)^a^	<0.001

Continuous variables are presented as mean ± SD or median (IQR); categorical variables as n (%) with non-missing denominators. Overall *P* values were obtained from one-way ANOVA, the Kruskal–Wallis test, or the chi-square test, as appropriate. Post-hoc comparisons. Where the overall test was significant, all six pairwise contrasts were tested: the Student–Newman–Keuls procedure for normally distributed continuous variables, Dunn’s test with Bonferroni adjustment for skewed variables, and pairwise chi-square tests with Bonferroni adjustment for categorical variables. Groups not sharing a superscript letter differ at the Bonferroni-adjusted two-sided α of 0.0083; for categorical variables the letters describe the distribution of the whole variable and are placed on the first level. Missing data: hypertension 6, BMI category 5, smoking 20, drinking 15, dyslipidemia 1, snoring 1, blood pressure 11, hs-CRP 250.

Overall, participants with worse glucose tolerance status tended to be older and had a less favourable cardiometabolic profile. Mean age, BMI, SBP, DBP, fasting glucose, 2-h postload glucose, TC, TG, LDL-C, and hs-CRP generally increased across glucose tolerance categories, whereas HDL-C tended to decrease. By contrast, eGFR did not differ significantly among the four groups. Pairwise *post-hoc* comparisons are indicated by superscript letters in **Table 2**; groups not sharing a letter differ at the Bonferroni-adjusted α of 0.0083. Two contrasts bear directly on the study hypothesis. First, 2-h postload glucose did not differ significantly between the NGT and IFG groups, whereas the IGT and diabetes groups were each distinct from these and from one another; fasting glucose, by contrast, separated all four groups. The IFG group in this cohort is therefore a phenotype of isolated fasting hyperglycaemia with essentially normal postload handling. Second, the prevalence of metabolic syndrome in the IFG (72.1%) and diabetes (77.2%) groups was statistically indistinguishable, and both were far above that in the NGT group (34.8%), whereas the IGT group (55.5%) differed from all three; this quantifies the extent to which metabolic syndrome overlaps with the fasting-glucose-defined phenotypes and motivates the modelling strategy set out in section 2.5.

The distributions of age group, education level, and BMI category also varied significantly across glucose tolerance groups. Participants with IGT and diabetes were more likely to be older and obese, whereas those with NGT were more often in the younger age category and had lower BMI. In addition, lower educational attainment was more common among participants with IGT and diabetes.

Regarding comorbidities, the prevalences of dyslipidaemia and metabolic syndrome increased substantially from the NGT group to the diabetes group: metabolic syndrome was present in 34.8% of the NGT group but in 55.5% of the IGT group and 77.2% of the diabetes group, an overlap with the exposure that motivated the modelling strategy described in section 2.5. As expected, diagnosed diabetes was observed exclusively in the diabetes group. Blood pressure also differed significantly across groups, with the lowest mean SBP and DBP in the NGT group (901 (68.3)% hypertensive) and the highest in the diabetes group (199 (87.3)%); the corresponding figures were 76 (89.4)% in the IFG group and 370 (82.6)% in the IGT group. In contrast, smoking status, drinking status, regular physical activity, snoring, sex distribution, and eGFR did not differ significantly across glucose tolerance groups.

### 3.3 Incidence of incident first-ever stroke and all-cause mortality

During follow-up, 121 participants developed incident first-ever post-baseline stroke and 68 died from any cause, corresponding to overall incidence rates of 9.25 and 5.05 per 1,000 person-years, respectively (**Table 3**).

**Table 3:** Crude and age- and sex-standardised incidence of incident first-ever stroke and all-cause mortality according to glucose tolerance status.

Outcome/Group	N	Events	Person-years	Crude incidence rate (per 1,000 PY)	Standardised incidence rate (95% CI, per 1,000 PY)
Incident first-ever stroke					
Normal glucose tolerance	1,305	63	8,348.64	7.55	5.83 (4.14–7.51)
IFG	85	3	555.83	5.40	6.41 (0.00–15.05)
IGT	441	35	2,748.25	12.74	10.38 (5.65–15.11)
Diabetes	226	20	1,422.14	14.06	19.85 (6.68–33.02)
Overall	2,057	121	13,074.86	9.25	7.73 (5.99–9.47)
All-cause mortality					
Normal glucose tolerance	1,305	40	8,554.03	4.68	4.56 (2.92–6.19)
IFG	85	1	565.94	1.77	1.88 (0.00–5.57)
IGT	441	18	2,870.79	6.27	4.09 (2.02–6.15)
Diabetes	226	9	1,486.64	6.05	3.49 (1.19–5.80)
Overall	2,057	68	13,477.40	5.05	4.16 (2.98–5.35)

Incident first-ever stroke was defined as the first stroke event occurring after the 2019 baseline examination; participants with a stroke before baseline were excluded. Person-time. Follow-up ran from the date of the baseline examination to the earliest of the event of interest, death, loss to follow-up, or the common end-of-follow-up date. For the stroke analysis, death without a preceding stroke censored follow-up; for the mortality analysis it was the event. This is why person-years differ between the two panels despite an identical analytic sample. All rates are given to two decimal places per 1,000 person-years. Standardisation. Rates were standardised for age (45–59, 60–69, ≥70 years) and sex by the direct method using the WHO world standard population, because the four groups differed significantly in age distribution (**Table 2**). 95% CIs were derived from the gamma distribution. PY, person-years; CI, confidence interval.

For incident first-ever stroke, the crude incidence rates were 7.55, 5.40, 12.74, and 14.06 per 1,000 person-years in the NGT, IFG, IGT, and diabetes groups, respectively. For all-cause mortality, the corresponding crude incidence rates were 4.68, 1.77, 6.27, and 6.05 per 1,000 person-years. After direct standardisation for age and sex, the stroke incidence rates were 5.83 (95% CI 4.14–7.51), 6.41 (0–15.05), 10.38 (5.65–15.11) and 19.85 (6.68–33.02) per 1,000 person-years in the NGT, IFG, IGT and diabetes groups respectively, so that the gradient across glucose tolerance categories was steeper after standardisation than before it. For all-cause mortality the standardised rates were 4.56 (2.92–6.19), 1.88 (0–5.57), 4.09 (2.02–6.15) and 3.49 (1.19–5.80) per 1,000 person-years; the crude excess seen in the IGT and diabetes groups therefore disappeared once age and sex were accounted for, which is consistent with the null mortality models reported below. Overall, both incident stroke and all-cause mortality occurred more frequently among participants with IGT and diabetes than among those with NGT.

### 3.4 Association between glucose tolerance status and incident first-ever stroke

The associations between glucose tolerance status and incident first-ever stroke are presented in **Table 4**. In Model 1 (unadjusted), both IGT and diabetes were associated with a higher risk of incident first-ever stroke compared with NGT, with HRs of 1.69 (95% CI: 1.11–2.55, *P* = 0.013) and 1.85 (95% CI: 1.12–3.06, *P* = 0.016), respectively. IFG was not associated with stroke risk (HR: 0.72, 95% CI: 0.22–2.28, *P* = 0.571).

**Table 4:** Associations of glucose tolerance status with incident first-ever stroke and all-cause mortality.

Glucose tolerance status	Model 1 HR (95% CI)	*P*	Model 2 HR (95% CI)	*P*	Model 3 HR (95% CI)	*P*
Incident first-ever stroke						
NGT (reference)	1.00	—	1.00	—	1.00	—
IFG	0.72 (0.22–2.28)	0.571	0.78 (0.25–2.51)	0.682	0.76 (0.24–2.45)	0.646
IGT	1.69 (1.11–2.55)	0.013	1.58 (1.03–2.41)	0.036 †	1.55 (1.01–2.39)	0.046 †
Diabetes	1.85 (1.12–3.06)	0.016	1.69 (1.02–2.81)	0.043 ‡	1.63 (0.96–2.79)	0.071 ‡
All-cause mortality						
NGT (reference)	1.00	—	1.00	—	1.00	—
IFG	0.38 (0.05–2.75)	0.337	0.39 (0.05–2.85)	0.354	0.46 (0.06–3.38)	0.443
IGT	1.34 (0.77–2.34)	0.301	1.00 (0.56–1.77)	0.987	1.05 (0.59–1.87)	0.875
Diabetes	1.29 (0.63–2.66)	0.491	1.01 (0.49–2.10)	0.977	1.19 (0.55–2.56)	0.662

All estimates are from Cox proportional hazards regression with NGT as the reference category; HRs are given to two decimal places and *P* values to three. Model 1: unadjusted. Model 2 (primary adjusted model): adjusted for sex, age group (45–59, 60–69, ≥70 years), smoking status, drinking status and eGFR. Model 3: Model 2 plus metabolic syndrome, which is reported separately because its diagnostic definition includes elevated fasting glucose or previously diagnosed diabetes and therefore overlaps with the exposure. **†** *P* = 0.046 in Model 3 is only marginally below the conventional threshold and should be read together with the power figures below. **‡** *P* = 0.075 is a borderline, non-significant result and is described as such throughout the text. Power. At α = 0.05 (two-sided) and 80% power, the accrued events allowed detection of hazard ratios of ≥1.94 for IGT (95 events), ≥2.40 for diabetes (81 events) and ≥4.34 for IFG (64 events) for stroke, and ≥2.36, ≥3.12 and ≥6.40 respectively for mortality. Proportional hazards were assessed by scaled Schoenfeld residuals and exposure-by-log(time) interaction terms (**Supplementary Table 2**). Complete-case sample: n = 2,031 (118 stroke events; 66 deaths) for Models 2 and 3.

In Model 2, the primary adjusted model (sex, age group, smoking, drinking, eGFR; n = 2,031 with 118 events), both intermediate and overt hyperglycaemia remained associated with incident first-ever stroke: the HR was 1.58 (95% CI: 1.03–2.41, *P* = 0.036) for IGT and 1.69 (95% CI: 1.02–2.81, *P* = 0.043) for diabetes, whereas IFG was not associated with risk (HR: 0.78, 95% CI: 0.25–2.51, *P* = 0.682). Adding metabolic syndrome (Model 3) attenuated both estimates: IGT remained significantly associated with stroke (HR: 1.55, 95% CI: 1.01–2.39, *P* = 0.046) but diabetes no longer reached significance (HR: 1.63, 95% CI: 0.96–2.79, *P* = 0.071), and IFG remained non-significant (HR: 0.76, 95% CI: 0.24–2.45, *P* = 0.646). This attenuation reflects the conceptual overlap between the exposure and the covariate rather than statistical instability: the maximum variance inflation factor across all Model 3 terms was 2.44, and the standard errors of the exposure coefficients rose by at most 4.9% between Models 2 and 3, while the coefficients themselves moved by 3.3% (IGT) and 6.4% (diabetes). Because the diagnostic definition of metabolic syndrome incorporates elevated fasting glucose or diagnosed diabetes, Model 3 removes part of the exposure itself, and Model 2 is therefore the estimate we interpret.

Kaplan–Meier analysis showed significant differences in stroke-free survival across glucose tolerance groups (log-rank χ² = 10.55, *P* = 0.014), with lower stroke-free survival probabilities in the IGT and diabetes groups than in the NGT group (**Figure 1**).

**Figure 1:**
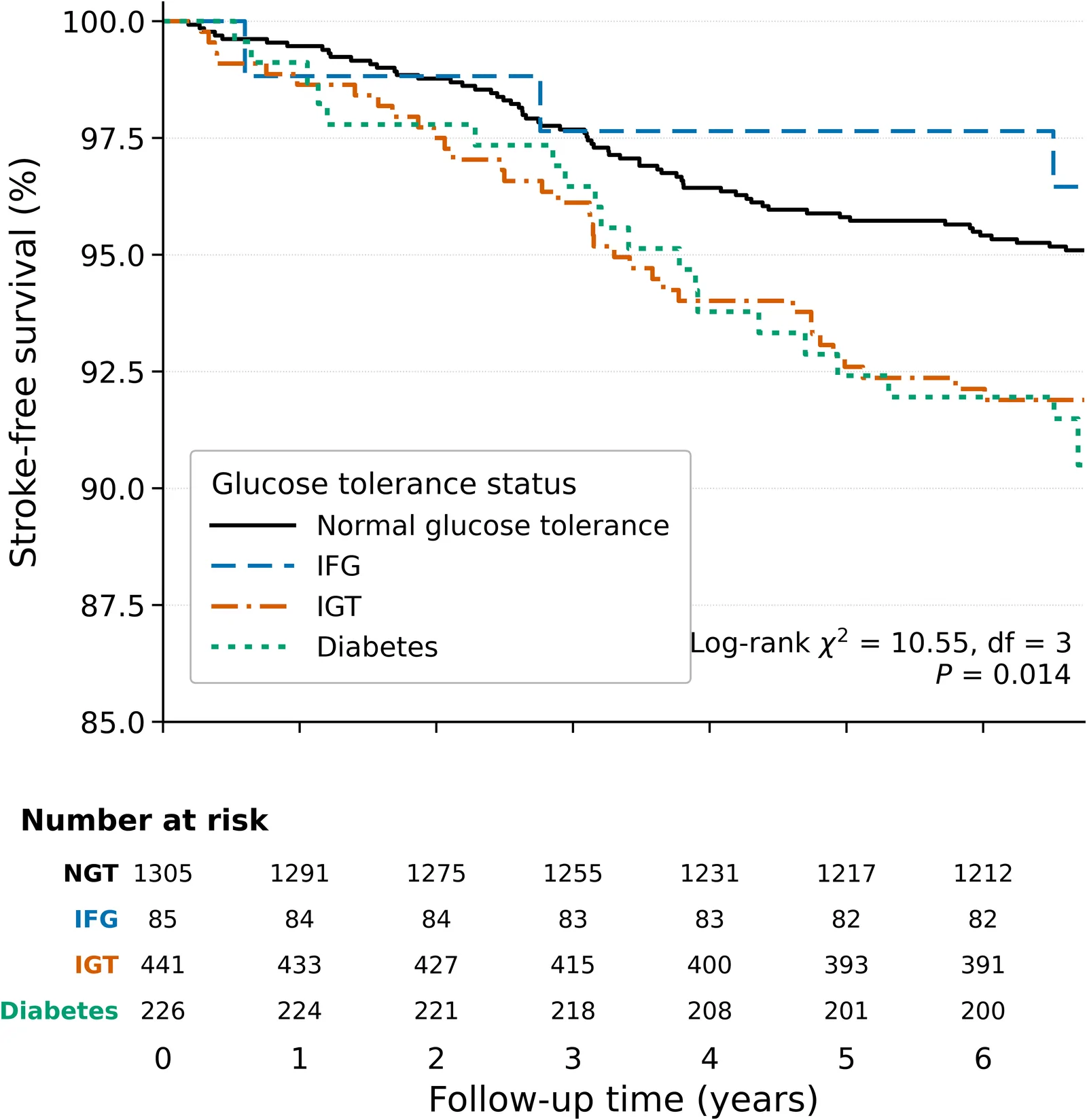
Kaplan–Meier curves for incident first-ever post-baseline stroke according to baseline glucose tolerance status. Kaplan–Meier estimates of stroke-free survival are shown for participants classified at baseline as having normal glucose tolerance (NGT), impaired fasting glucose (IFG), impaired glucose tolerance (IGT), or diabetes. Analytic sample: 2,057 participants (NGT 1,305; IFG 85; IGT 441; diabetes 226) contributing 13,074.9 person-years, with 121 incident events over a maximum follow-up of the maximum observed follow-up. Incident stroke was defined as the first stroke event occurring after the 2019 baseline examination; participants with a stroke before baseline were excluded. Follow-up was censored at death without stroke, at loss to follow-up, or at the common end-of-follow-up date, whichever came first. The between-group difference was statistically significant (log-rank χ² = 10.55, df = 3, *P* = 0.014). Participants with IGT and diabetes showed lower stroke-free survival probabilities during follow-up than those with NGT, whereas the IFG group did not show a clear excess risk pattern. The number of participants remaining at risk in each group at each year of follow-up is shown beneath the horizontal axis.

### 3.5 Association between glucose tolerance status and all-cause mortality

The associations between glucose tolerance status and all-cause mortality are also shown in **Table 4**. No significant association was observed between baseline glucose tolerance status and all-cause mortality in any model. In Model 1, the HRs were 0.38 (95% CI: 0.05–2.75, *P* = 0.337) for IFG, 1.34 (95% CI: 0.77–2.34, *P* = 0.301) for IGT, and 1.29 (95% CI: 0.63–2.66, *P* = 0.491) for diabetes, compared with NGT.

In Model 2 the adjusted HRs were 0.39 (95% CI: 0.05–2.85, *P* = 0.354) for IFG, 1.00 (95% CI: 0.56–1.77, *P* = 0.987) for IGT and 1.01 (95% CI: 0.49–2.10, *P* = 0.977) for diabetes; in Model 3 the adjusted HRs were 0.46 (95% CI: 0.06–3.38, *P* = 0.443) for IFG, 1.05 (95% CI: 0.59–1.87, *P* = 0.875) for IGT, and 1.19 (95% CI: 0.55–2.56, *P* = 0.662) for diabetes.

Kaplan–Meier curves for all-cause mortality showed only modest differences across glucose tolerance groups, and the between-group difference was not statistically significant (log-rank χ² = 2.58, *P* = 0.461) (**Figure 2**). The contrast with **Figure 1** is therefore substantive rather than presentational: the four survival curves separate clearly for stroke but not for death, and the two outcomes are described separately throughout.

**Figure 2:**
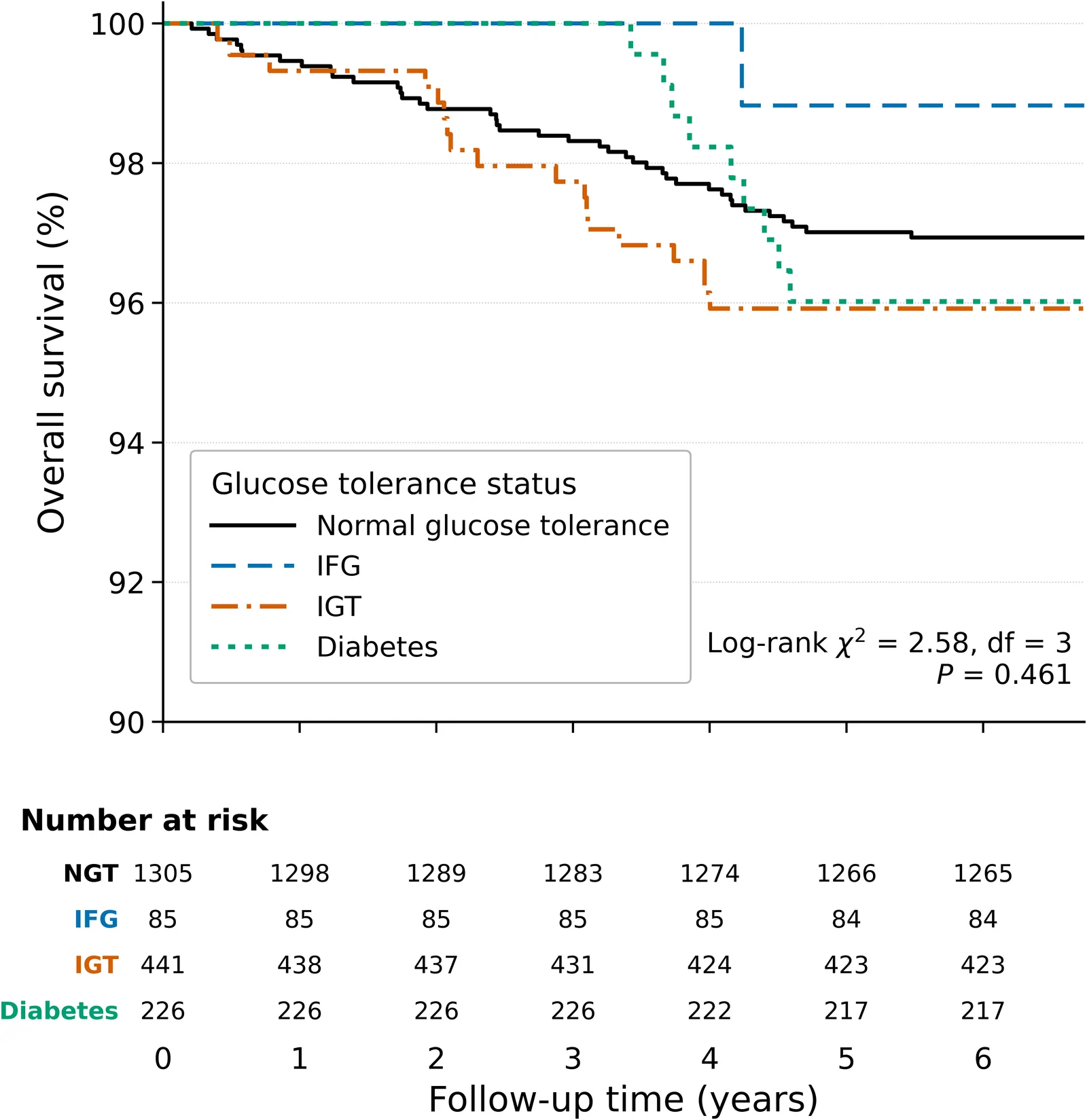
Kaplan–Meier curves for all-cause mortality according to baseline glucose tolerance status. Kaplan–Meier estimates of overall survival are shown for participants classified at baseline as having NGT, IFG, IGT, or diabetes. Analytic sample: 2,057 participants contributing 13,477.4 person-years, with 68 deaths; follow-up was censored at loss to follow-up or at the common end-of-follow-up date, and an intervening stroke did not censor follow-up. The between-group difference was not statistically significant (log-rank χ² = 2.58, df = 3, *P* = 0.461). In contrast to **Figure 1**, the four survival curves remained closely superimposed throughout follow-up, consistent with the null associations observed in the Cox models. The number of participants remaining at risk in each group at each year of follow-up is shown beneath the horizontal axis.

### 3.6 Associations of continuous glucose indicators with study outcomes

The associations of continuous glucose indicators with incident first-ever stroke and all-cause mortality are presented in **Table 5**. Each glucose measure was entered into a separate model, because the two were strongly correlated in this cohort (Pearson *r* = 0.66) and joint entry would render the individual coefficients uninterpretable. Both measures were associated with incident first-ever stroke. The HR was 1.18 per 1 mmol/L of fasting glucose (95% CI: 1.01–1.38, *P* = 0.035) and 1.07 per 1 mmol/L of 2-h postload glucose (95% CI: 1.02–1.13, *P* = 0.008). Because a 1 mmol/L increment means something different for the two measures, the estimates are also expressed per standard deviation of the cohort distribution (0.92 mmol/L for fasting and 2.87 mmol/L for postload glucose): on that common scale the postload association was the stronger of the two, at 1.23 (95% CI: 1.05–1.42) versus 1.17 (95% CI: 1.01–1.35) per standard deviation.

**Table 5:** Associations of continuous glucose indicators with incident first-ever stroke and all-cause mortality.

Exposure	N	Events	HR per 1 mmol/L (95% CI)	*P*	HR per 1 SD	*P*
Incident first-ever stroke						
Fasting glucose	2,031	118	1.18 (1.01–1.38)	0.035	1.17 (1.01–1.35)	0.035
2-h postload glucose	2,031	118	1.07 (1.02–1.13)	0.008	1.23 (1.05–1.42)	0.008
All-cause mortality						
Fasting glucose	2,031	66	1.05 (0.81–1.35)	0.718	1.04 (0.83–1.32)	0.718
2-h postload glucose	2,031	66	1.02 (0.94–1.10)	0.691	1.05 (0.83–1.32)	0.691

Fasting glucose and 2-h postload glucose were entered into separate Cox models — never simultaneously — because the two were strongly correlated in this cohort (Pearson *r* = 0.66) and joint entry would render the individual coefficients uninterpretable. Each model was adjusted for the Model 2 covariates (sex, age group, smoking status, drinking status, eGFR); metabolic syndrome was omitted because its fasting-glucose component is collinear with the fasting-glucose exposure. Per-SD hazard ratios were obtained by rescaling the per-unit coefficient by the cohort standard deviation (0.92 mmol/L fasting, 2.87 mmol/L postload); the *P* values are therefore identical to the per-unit values. On this common scale the postload association with stroke is the stronger of the two. The analytic sample is the same complete-case set used for Models 2 and 3 in **Table 4** (n = 2,031; 118 stroke events, 66 deaths). HR, hazard ratio; CI, confidence interval; SD, standard deviation.

The continuous and categorical analyses should be read together, because they are not simply two views of the same result. The continuous fasting association coexists with a null estimate for the IFG category, and the two are reconciled by how the categories are constructed: participants with fasting glucose of 7.0 mmol/L or above are classified as diabetes rather than IFG, so the IFG group contains only the narrow band from 6.1 to 6.9 mmol/L with normal postload values, whereas the continuous fasting variable spans the full range including the diabetes group. The continuous fasting association is therefore driven substantially by participants in the diabetes range, and it is not evidence that isolated fasting hyperglycaemia carries excess stroke risk in this population. The postload signal, by contrast, is concordant across the two analyses: 2-h glucose was associated with stroke as a continuous variable, and the postload phenotype (IGT) was associated with stroke as a category.

In clinical terms, each 1 mmol/L higher 2-h postload glucose corresponded to a 7% higher hazard of first-ever stroke, so a participant at the upper limit of the IGT range (11.0 mmol/L) would carry an estimated 23% higher hazard than one at 8.0 mmol/L, if the association were causal — an inference the present data do not establish.

For all-cause mortality, neither fasting glucose nor 2-h postload glucose was significantly associated with risk after adjustment. The adjusted HRs were 1.05 (95% CI: 0.81–1.35, *P* = 0.718) for fasting glucose and 1.02 (95% CI: 0.94–1.10, *P* = 0.691) for 2-h postload glucose.

### 3.7 Proportional hazards assumption and sensitivity analyses

For the incident-stroke model the proportional hazards assumption was satisfied for the exposure and for every covariate: the global test on scaled Schoenfeld residuals gave χ² = 4.10 (df = 11, *P* = 0.967) and no individual term reached *P* < 0.13, while the log(−log(survival)) plots showed approximately parallel curves throughout follow-up. For the mortality model the global test was also non-significant (χ² = 15.25, df = 11, *P* = 0.171), but two individual terms did show evidence of time-varying effects — the diabetes indicator (*P* = 0.016) and eGFR (*P* = 0.034). Since all mortality estimates were null and remained so in every specification, we report this departure rather than reinterpreting it, and note in the Limitations that the mortality hazard ratios should be read as averages over follow-up. Full results are given in **Supplementary Table 2**.

Sensitivity analyses are summarised in **Table 6**. Multiple imputation of missing covariates (20 imputed datasets, all 2,057 eligible participants) gave an adjusted HR for IGT versus NGT of 1.59 (95% CI: 1.04–2.41, *P* = 0.031), against 1.58 (95% CI: 1.03–2.41) in the complete-case analysis, and 1.65 (95% CI: 1.00–2.74, *P* = 0.052) for diabetes; the exclusion of the 26 participants with missing covariates therefore did not materially bias the estimates. In the Fine–Gray model treating death without stroke as a competing event (57 competing events), the subdistribution HRs were 1.57 (95% CI: 1.02–2.43, *P* = 0.043) for IGT and 1.72 (95% CI: 1.03–2.86, *P* = 0.038) for diabetes. The close agreement with the cause-specific estimates is consistent with the low absolute mortality in this cohort (68 deaths, 5.05 per 1,000 person-years), which leaves limited scope for competing risk to distort the cumulative incidence of stroke. Additional adjustment for systolic blood pressure, BMI and LDL-C attenuated both estimates below significance (IGT: HR 1.42, 95% CI: 0.92–2.18, *P* = 0.111; diabetes: HR 1.37, 95% CI: 0.81–2.30, *P* = 0.242). We report this specification as a check on residual confounding rather than as an alternative primary model, because these three variables are treated as mediators in the assumed causal structure (section 2.5); the attenuation is what adjustment for a mediator would produce, but it also shows that the findings are not robust to the opposite causal assumption.

**Table 6:** Sensitivity analyses for the association between glucose tolerance status and incident first-ever stroke.

Analysis	IFG vs NGT HR (95% CI)	IGT vs NGT HR (95% CI)	Diabetes vs NGT HR (95% CI)	Purpose
Model 2 (primary; no metabolic syndrome)	0.78 (0.25–2.51)	1.58 (1.03–2.41)	1.69 (1.02–2.81)	Reference for comparison
Model 3 (+ metabolic syndrome)	0.76 (0.24–2.45)	1.55 (1.01–2.39)	1.63 (0.96–2.79)	Effect of adding metabolic syndrome
Multiple imputation (m = 20), Model 2 covariates	0.76 (0.24–2.42)	1.59 (1.04–2.41)	1.65 (1.00–2.74)	Tests the complete-case assumption
Fine–Gray subdistribution hazard (death without stroke as competing event)	0.80 (0.25–2.58)	1.57 (1.02–2.43)	1.72 (1.03–2.86)	Tests competing-risk distortion
Additionally adjusted for SBP, BMI and LDL-C	0.65 (0.20–2.09)	1.42 (0.92–2.18)	1.37 (0.81–2.30)	Tests residual vascular confounding

All models use NGT as the reference category. Model 2 covariates are sex, age group, smoking status, drinking status and eGFR. Multiple imputation used chained equations with predictive mean matching for continuous variables, including all model covariates, the Nelson–Aalen cumulative hazard estimate and the event indicator in the imputation model; estimates were pooled by Rubin’s rules. The Fine–Gray model estimates subdistribution hazard ratios for the cumulative incidence of stroke in the presence of death as a competing event.

### 3.8 Subgroup analyses

Prespecified subgroup analyses of incident first-ever stroke by age, sex and metabolic syndrome status are shown in **Table 7**. No interaction test reached significance. The IGT–stroke HR was 3.11 (95% CI: 0.87–11.05) among participants aged under 60 years and 1.45 (95% CI: 0.92–2.27) among those aged 60 years or older, and the corresponding diabetes–stroke HRs were 7.36 (95% CI: 2.24–24.16) and 1.34 (95% CI: 0.76–2.37); the likelihood ratio test for interaction gave *P* = 0.081. Estimates were similar in men and women (IGT: 1.64 versus 1.43; *P* for interaction = 0.779) and in participants with and without metabolic syndrome (IGT: 1.47 versus 1.61; *P* for interaction = 0.972). The apparently large relative risks in the under-60 stratum rest on only 16 events and correspondingly wide confidence intervals, and the interaction test does not support a genuine difference by age; these estimates are exploratory and should not be interpreted as evidence of effect modification.

**Table 7:** Prespecified subgroup analyses of the association between glucose tolerance status and incident first-ever stroke.

Subgroup	n/events	IFG vs NGT	IGT vs NGT	Diabetes vs NGT	*P* for interaction
Age					0.081
<60 years	631/16	2.35 (0.28–19.65)	3.11 (0.87–11.05)	7.36 (2.24–24.16)	
≥60 years	1,400/102	0.59 (0.14–2.42)	1.45 (0.92–2.27)	1.34 (0.76–2.37)	
Sex					0.779
Men	893/70	0.43 (0.06–3.17)	1.64 (0.93–2.87)	1.84 (0.97–3.49)	
Women	1,138/48	1.39 (0.33–5.91)	1.43 (0.74–2.74)	1.49 (0.64–3.50)	
Metabolic syndrome					0.972
Present	924/55	0.72 (0.17–3.09)	1.47 (0.77–2.81)	1.67 (0.85–3.27)	
Absent	1,107/63	0.93 (0.13–6.80)	1.61 (0.90–2.88)	1.41 (0.55–3.65)	

Models within each stratum were adjusted for the Model 2 covariates other than the stratifying variable itself. *P* for interaction was obtained from a likelihood ratio test comparing models with and without a multiplicative interaction term between glucose tolerance status and the stratifying variable.

## 4 Discussion

### 4.1 Main findings

In this low-income rural Chinese cohort followed for a median of 6.7 years, three findings emerged. Both IGT and diabetes were associated with an increased risk of incident first-ever stroke in the primary adjusted model, whereas isolated IFG was not. Glucose tolerance status was not associated with all-cause mortality in any model. In continuous analyses both glucose measures predicted stroke, but postload glycaemia carried the stronger association per standard deviation, and it was the only one whose categorical counterpart was also associated with risk.

### 4.2 Glucose tolerance status and incident first-ever stroke

Whether abnormal glucose tolerance, particularly prediabetic phenotypes, is independently associated with future stroke has not been fully settled, and the relative importance of IGT versus IFG remains a matter of ongoing debate. In a meta-analysis of prospective studies, Lee et al. found that prediabetes was associated with an increased risk of future stroke when defined by IGT or by combined criteria including IGT, whereas isolated IFG was not significantly associated with stroke risk (9). Consistent with this pattern, Hyvärinen et al. reported that hyperglycaemia defined by 2-hour glucose criteria was more strongly associated with stroke incidence than fasting-based criteria (22). Similarly, Wang et al. showed in a large Chinese cohort that postload plasma glucose had greater predictive value for cardiovascular disease than fasting plasma glucose (23). By contrast, some studies have suggested that fasting glucose may also predict stroke risk under certain conditions (15), and diabetes has been consistently linked to excess future stroke risk in Chinese adults (24). Our findings are broadly in line with the view that postload-related dysglycaemia is more relevant to stroke than isolated fasting abnormality.

Four mechanisms plausibly underlie this asymmetry. First, IGT is by definition a disorder of postprandial glucose handling, and postprandial excursions — as opposed to a stable elevation of fasting glucose — expose the endothelium to repeated, transient glucose peaks. Acute hyperglycaemia suppresses endothelial nitric oxide availability, increases mitochondrial superoxide production and activates the polyol, hexosamine and protein kinase C pathways, so that glycaemic variability generates oxidative and inflammatory stress that a comparable mean glucose held constant does not (22, 23). Second, IGT and IFG are not two grades of one abnormality but two different physiological lesions: IGT reflects predominantly peripheral (skeletal muscle) insulin resistance together with impaired late-phase insulin secretion, whereas isolated IFG reflects predominantly hepatic insulin resistance and impaired first-phase secretion. Peripheral insulin resistance is the phenotype more tightly coupled to endothelial dysfunction, and recent Chinese cohort evidence linking a lower estimated glucose disposal rate — a marker of peripheral insulin sensitivity — to higher stroke risk supports this reading (25). This offers a coherent explanation for why only IGT was associated with stroke here. Third, the dietary structure of this population is relevant to the magnitude of the effect: the staple diet in rural northern China is dominated by refined cereal starch with comparatively low protein and fat content per meal, a pattern that produces large postprandial glucose excursions. Participants classified as IGT in this setting may therefore experience greater cumulative postprandial glycaemic exposure than an IGT classification would imply in a population with a lower-glycaemic-load diet, which would tend to strengthen the IGT–stroke association specifically in cohorts such as this one. Fourth, IGT may act partly as a marker of a broader vulnerable metabolic phenotype in a population with clustered and poorly controlled vascular risk factors, rather than as an isolated causal exposure.

How our estimates compare with the wider literature is worth setting out explicitly. The DECODE Study, in 22 European cohorts with more than 25,000 participants, found that 2-h glucose predicted cardiovascular mortality after adjustment for fasting glucose, whereas fasting glucose added little after adjustment for 2-h glucose (10, 11). Our directional finding agrees, but our confidence intervals are far wider, reflecting a cohort two orders of magnitude smaller. The Da Qing studies, which followed participants with IGT for up to 34 years, reported that roughly half of Chinese adults with IGT or newly diagnosed diabetes eventually sustained a stroke (26); our six-year follow-up captures only the earliest portion of that trajectory, which is consistent with our smaller point estimates. Lee et al.’s meta-analysis reported a pooled relative risk of about 1.2 for stroke in prediabetes defined by IGT (9), somewhat lower than our HR of 1.58, a difference attributable in part to the higher background vascular risk of this population and in part to the imprecision discussed below. The three principal sources of divergence between studies are therefore population risk profile, follow-up duration and sample size, and our study sits at the small-sample, short-follow-up, high-background-risk corner of that space.

A caution about magnitude is warranted. As set out in section 2.5, the study had 80% power to detect an IGT–stroke HR of 1.94 or greater and a diabetes–stroke HR of 2.40 or greater, so observed HRs of 1.58 and 1.69 that only just cross the significance threshold are likely to overstate the true effects — the winner’s curse familiar from underpowered studies. What supports the direction of these findings is their consistency rather than any single *P* value: the categorical and continuous analyses agree, the multiple-imputation and competing-risk analyses reproduce the estimates closely, and the direction matches the prior literature. Their magnitude should not be taken at face value.

### 4.3 Glucose tolerance status and all-cause mortality

Meta-analytic evidence suggests that prediabetes is associated with a modest increase in all-cause mortality in the general population (8). Long-term follow-up data from the Da Qing studies further demonstrated that diabetes and IGT were associated with excess mortality over decades of follow-up, and that lifestyle intervention in individuals with IGT reduced long-term mortality risk (12–14). More recently, national data from China have shown that both diabetes and prediabetes are associated with increased mortality and reduced life expectancy (7). However, cohort studies in rural Chinese populations have also suggested more complex patterns, including possible non-linear associations between fasting glucose and mortality (16).

In contrast, we did not observe significant associations between glucose tolerance status and all-cause mortality. Four explanations deserve consideration, and they are not mutually exclusive.

First, follow-up duration. Six years is short relative to the latency over which dysglycaemia translates into excess total mortality. The Da Qing cohort required 23 to 30 years of observation before mortality differences by baseline glucose tolerance became clearly established (13, 14), and its 34-year analysis showed that stroke events accumulate throughout, but that survival curves separate late (26). A null mortality finding at six years is therefore what the Da Qing trajectory would predict, not a contradiction of it.

Second, statistical power. Only 68 deaths occurred. As quantified in section 2.5, this yields 80% power only for HRs of 2.36 (IGT), 3.12 (diabetes) and 6.40 (IFG) versus NGT — effect sizes far larger than any reported in the literature for these exposures. The mortality analysis was thus incapable of detecting a plausible effect, and the null result carries very little evidential weight.

Third, competing causes of death. In rural Chinese populations a substantial share of deaths is attributable to infection, digestive and respiratory disease, malignancy and injury — causes with little or no relation to glycaemic status. Where such causes constitute a large fraction of total mortality, any glycaemia-attributable excess is diluted within the composite endpoint, and all-cause mortality becomes an insensitive instrument for detecting it. Cause-specific mortality would be the more informative endpoint, but cause of death could not be reliably classified in this community-surveillance setting.

Fourth, and consistent with the above, the vascular consequences of dysglycaemia in this population appear earlier in cerebrovascular events than in total survival. This ordering — stroke first, mortality later — is the pattern the Da Qing long-term data describe, and our results are compatible with an early position on that same trajectory rather than with an absence of effect.

### 4.4 Continuous glucose indicators: postload versus fasting glycaemia

Evidence from the DECODE Study and related analyses suggests that 2-h glucose may be more predictive of cardiovascular outcomes than fasting glucose (10, 11). In particular, postload glucose has been linked more closely to stroke-related outcomes in several cohort studies (22, 23, 27), whereas fasting glucose has shown less consistent associations (15, 16). In patients with coronary artery disease, Shahim et al. also reported that 2-h postload glucose showed stronger prognostic value than fasting glucose for adverse outcomes (28). Our findings are directionally consistent with this body of work: fasting glucose was not associated with either outcome, whereas 2-h postload glucose showed a positive association with incident first-ever stroke, and the same asymmetry appeared in the categorical analysis, where IGT but not IFG was associated with stroke.

Three qualifications matter here. First, the fasting association should not be read as evidence that isolated fasting hyperglycaemia raises stroke risk in this population: because participants with fasting glucose at or above 7.0 mmol/L are classified as diabetes, the continuous fasting variable spans the diabetes range while the IFG category does not, and the categorical analysis found no excess risk in IFG. Second, the postload advantage, although consistent across the categorical and continuous analyses and larger per standard deviation, is a difference in degree rather than a demonstration that one measure can replace the other; we did not test whether adding 2-h glucose to a model containing fasting glucose improves discrimination, calibration or reclassification, and the number of events here would not support such a test. Third, the estimates rest on 118 events in an analysis powered only for larger effects, and they lost significance on additional adjustment for blood pressure, BMI and lipids. What these data support is that postload dysglycaemia is prognostically relevant to stroke in this population and that it deserves direct evaluation. However, the findings of this study still require further experimental validation.

### 4.5 Clinical and public health implications

Three implications follow, each stated at the level of confidence the data will bear.

Clinically, risk assessment for stroke in middle-aged and older rural adults should not rest solely on the presence or absence of diagnosed diabetes. Individuals with normal fasting glucose but postload values in the IGT range are, on the present evidence, a group whose vascular risk may be underestimated by fasting measurement alone. Where an OGTT is feasible, it may identify such individuals; whether doing so changes outcomes is untested.

For public health practice, the more defensible reading is that IGT is a candidate target for evaluation rather than an established screening indication. A staged approach would be to add postload measurement to existing chronic-disease examinations for a defined higher-risk subgroup, and to evaluate the yield and the downstream effect of lifestyle intervention in those identified — an intervention for which the Da Qing trial provides direct randomised evidence of long-term benefit in IGT (12–14).

At the policy level, our findings do not justify a recommendation to reallocate scarce resources toward universal postload screening in resource-limited areas. The OGTT is substantially more costly in staff time and participant burden than fasting measurement, and the incremental prognostic value demonstrated here is neither large nor statistically secure. The appropriate next step is a formal evaluation of incremental predictive performance and cost-effectiveness, not a change in practice.

### 4.6 Strengths

This study has five specific strengths. First, it is drawn from a prospective, community-based cohort in a low-income rural area with continuous stroke surveillance since 1985 — a population that carries a high vascular burden yet is markedly underrepresented in the epidemiological literature. Second, all participants underwent a standard 75-g OGTT, permitting complete four-way glucose tolerance phenotyping (NGT, IFG, IGT, diabetes) rather than the dichotomous classification used in most cohorts, and therefore allowing IFG and IGT to be separated. Third, stroke and all-cause mortality were evaluated within a single analytical framework, so that the divergence between the two outcomes can be interpreted directly rather than inferred across studies. Fourth, glycaemia was analysed both categorically and as two separate continuous measures, and the concordance between these two approaches strengthens the internal consistency of the postload signal. Fifth, the models adjust for renal function alongside demographic and lifestyle confounders, and the effect of metabolic confounding is examined explicitly rather than assumed.

### 4.7 Limitations

Several limitations should be acknowledged. First, this was an observational cohort study conducted in a specific low-income rural population in northern China; causal inference remains limited, and residual confounding cannot be excluded. In particular, the primary model does not adjust for blood pressure, BMI, lipid fractions or physical activity. These were omitted deliberately, on the reasoning that they lie on the causal pathway between insulin resistance and stroke, but that reasoning depends on an assumed causal structure that cannot be verified from these data. This is not a hypothetical concern: when systolic blood pressure, BMI and LDL-C were added to the model, the IGT and diabetes estimates fell to 1.42 and 1.37 and neither remained significant (**Table 6**). If those variables are confounders rather than mediators, the primary estimates are biased upward and the association with stroke is weaker than reported.

Second, metabolic syndrome overlaps conceptually with the exposure, because its diagnostic definition includes elevated fasting glucose or diagnosed diabetes. We addressed this by excluding it from the primary model and reporting it separately, but the possibility of residual overadjustment or collinearity in Model 3 remains.

Third, the primary analyses were complete-case, which is unbiased only under a missing-completely-at-random assumption. Multiple imputation gave concordant results, but a mechanism in which missingness depends on unmeasured determinants of both exposure and outcome cannot be excluded, so selection bias cannot be ruled out.

Fourth, although events were adjudicated by neurologists blinded to exposure, imaging confirmation was not available for every case, and stroke subtype could not be determined in all events; some outcome misclassification is therefore possible. Non-fatal strokes that never came to medical attention would not have been captured.

Fifth, statistical power was limited. The study could not detect HRs below 1.94 for IGT–stroke, below 2.40 for diabetes–stroke or below 4.34 for IFG–stroke at 80% power, and the mortality analyses were weaker still. The IFG group (n = 86) is too small to support any conclusion, and the significant IGT result is likely to overstate the true effect size.

Sixth, death is a competing event for incident stroke. The Fine–Gray sensitivity analysis gave estimates close to the cause-specific model, but competing-risk effects cannot be entirely excluded.

Seventh, the proportional hazards assumption held throughout the stroke models, but in the mortality models the diabetes indicator (*P* = 0.016) and eGFR (*P* = 0.034) showed time-varying effects, so the mortality hazard ratios should be read as averages over follow-up rather than as constant effects. Tests of this kind also have limited power with 121 and 68 events, so modest non-proportionality elsewhere may have gone undetected.

Eighth, incident stroke was defined as the first post-baseline event, and recurrent stroke events were not included; our findings therefore reflect first stroke risk rather than the total burden of recurrent cerebrovascular events. Ninth, glucose tolerance status was classified from baseline measurements only, and subsequent changes in glucose metabolism during follow-up could not be taken into account; nor was the OGTT repeated to confirm classification, so single-measurement misclassification is possible and would be expected to attenuate the observed associations toward the null. Tenth, other potentially informative glycaemic indicators, such as HbA1c or glycaemic variability, were not available. Finally, generalisability is limited: the findings apply most directly to rural northern Chinese communities of comparable income level, dietary pattern and primary-care infrastructure, and should not be extrapolated to urban Chinese populations, to hospital cohorts, or to populations with substantially different dietary or health-service contexts.

## 5 Conclusion

In this population-based prospective cohort of low-income rural Chinese adults, both IGT and diabetes were associated with an increased risk of incident first-ever post-baseline stroke, whereas isolated IFG was not, and glucose tolerance status was not associated with all-cause mortality over a median of 6.7 years. Continuous analyses pointed in the same direction, with postload glycaemia carrying the stronger association per standard deviation. Given the limited number of events, these results are best read as identifying postload dysglycaemia — and the IGT phenotype in particular — as a target worth evaluating prospectively in disadvantaged rural populations, not as evidence that postload glucose screening should be adopted.

## Data Availability

The raw data supporting the conclusions of this article will be made available by the authors, without undue reservation.

## Supplementary material

Supplementary Table 1 Baseline characteristics of participants included in versus excluded from the time-to-event analyses. Values are mean ± SD, median (IQR) or n (%). The excluded participants are the 30 individuals without a valid baseline examination date, who could not contribute person-time. Continuous variables were compared using Welch’s *t*-test or the Mann–Whitney *U* test, categorical variables using the chi-square test. The two groups were similar in age, sex, adiposity, blood pressure, both glucose measures, renal function, lipid profile, hs-CRP, metabolic syndrome, diagnosed diabetes and — most importantly for the present analysis — the distribution of glucose tolerance status (*P* = 0.819). Excluded participants had a lower prevalence of hypertension and a higher prevalence of current smoking; with 30 excluded individuals and 16 comparisons, neither difference survives Bonferroni adjustment (α = 0.003), and both are compatible with chance. Exclusion is therefore unlikely to have introduced material selection bias with respect to the exposure.

Supplementary Table 2 Tests of the proportional hazards assumption. χ² is the test statistic for the correlation between scaled Schoenfeld residuals and log-transformed survival time, aggregated across the indicator variables making up each term. *P* < 0.05 indicates evidence against the proportional hazards assumption. Exposure-by-log(time) interaction terms were additionally fitted for each outcome.

Supplementary Figure 1 Flow of participants through the study. STROBE-style flow diagram showing the 2,087 participants who attended the 2019 baseline examination with a complete 75-g oral glucose tolerance test and without a stroke documented before baseline, the 30 excluded for lack of a valid baseline examination date, the 2,057 entering the time-to-event analyses of both outcomes, and the complete-case samples for the multivariable models.
