# Supplementary material for: Glucose tolerance phenotypes and risks of incident first-ever stroke and all-cause mortality in low-income rural Chinese adults: a population-based prospective cohort study
Source: Front Endocrinol (Lausanne). 2026 Sep 17;17:1924067. doi: 10.3389/fendo.2026.1924067 (PMC13627055; doi:10.3389/fendo.2026.1924067)
Supplement: Supplementary file 1 [file Table1.docx]

**Supplementary Table S1. Baseline characteristics of participants included in versus excluded from the time-to-event analyses**

| Characteristic | Included (n = 2,057) | Excluded (n = 30) | *P* |
| --- | --- | --- | --- |
| Age, years | 63.41 ± 7.89 | 64.66 ± 7.06 | 0.342 |
| Men, n (%) | 908 (44.1) | 18 (60.0) | 0.121 |
| BMI, kg/m² | 25.61 ± 3.67 | 25.38 ± 4.40 | 0.775 |
| SBP, mmHg | 149.08 ± 19.67 | 148.76 ± 22.66 | 0.940 |
| DBP, mmHg | 85.16 ± 10.69 | 86.60 ± 14.60 | 0.601 |
| Fasting glucose, mmol/L | 5.54 ± 0.92 | 5.42 ± 0.63 | 0.284 |
| 2-h postload glucose, mmol/L | 7.28 ± 2.87 | 7.24 ± 2.63 | 0.946 |
| eGFR, mL/min/1.73 m² | 87.93 ± 11.84 | 85.87 ± 14.16 | 0.435 |
| TC, mmol/L | 5.14 ± 0.87 | 5.20 ± 0.90 | 0.718 |
| hs-CRP, mg/L | 1.07 (0.62–2.15) | 1.16 (0.73–3.34) | 0.302 |
| Hypertension, n (%) | 1530 (74.6) | 16 (53.3) | 0.015 |
| Metabolic syndrome, n (%) | 935 (45.5) | 13 (43.3) | 0.963 |
| Diagnosed diabetes, n (%) | 226 (11.0) | 2 (6.7) | 0.647 |
| Current smoking, n (%) | 512 (25.1) | 14 (46.7) | 0.013 |
| Current drinking, n (%) | 419 (20.5) | 6 (20.0) | 1.000 |
| Glucose tolerance status, n (%) |  |  | 0.819 |
| NGT | 1305 (63.4) | 19 (63.3) |  |
| IFG | 85 (4.1) | 1 (3.3) |  |
| IGT | 441 (21.4) | 8 (26.7) |  |
| Diabetes | 226 (11.0) | 2 (6.7) |  |

Values are mean ± SD, median (IQR) or n (%). The excluded participants are the 30 individuals without a valid baseline examination date, who could not contribute person-time. Continuous variables were compared using Welch's *t*-test or the Mann–Whitney *U* test, categorical variables using the chi-square test. The two groups were similar in age, sex, adiposity, blood pressure, both glucose measures, renal function, lipid profile, hs-CRP, metabolic syndrome, diagnosed diabetes and — most importantly for the present analysis — the distribution of glucose tolerance status (*P* = 0.819). Excluded participants had a lower prevalence of hypertension and a higher prevalence of current smoking; with 30 excluded individuals and 16 comparisons, neither difference survives Bonferroni adjustment (α = 0.003), and both are compatible with chance. Exclusion is therefore unlikely to have introduced material selection bias with respect to the exposure.

**Supplementary Table S2. Tests of the proportional hazards assumption**

| Term | Incident stroke χ² | *P* | All-cause mortality χ² | *P* |
| --- | --- | --- | --- | --- |
| Glucose tolerance status (df = 3) | 0.49 | 0.921 | 6.75 | 0.080 |
| Sex (df = 1) | 0.00 | 0.979 | 0.93 | 0.334 |
| Age group (df = 2) | 2.51 | 0.286 | 1.88 | 0.390 |
| Smoking status (df = 2) | 0.52 | 0.772 | 0.52 | 0.770 |
| Drinking status (df = 2) | 0.33 | 0.846 | 0.68 | 0.712 |
| eGFR (df = 1) | 0.26 | 0.614 | 4.49 | 0.034 |
| GLOBAL (df = 11) | 4.10 | 0.967 | 15.25 | 0.171 |

χ² is the test statistic for the correlation between scaled Schoenfeld residuals and log-transformed survival time, aggregated across the indicator variables making up each term. *P* < 0.05 indicates evidence against the proportional hazards assumption. Exposure-by-log(time) interaction terms were additionally fitted for each outcome.
